# Application of Spatial and Closed Capture-Recapture Models on Known Population of the Western Derby Eland (*Taurotragus derbianus derbianus*) in Senegal

**DOI:** 10.1371/journal.pone.0136525

**Published:** 2015-09-03

**Authors:** Tomáš Jůnek, Pavla Jůnková Vymyslická, Kateřina Hozdecká, Pavla Hejcmanová

**Affiliations:** 1 Department of Ecology, Faculty of Environmental Sciences, Czech University of Life Sciences Prague, Kamýcká 129, 165 21 Prague 6 –Suchdol, Prague, Czech Republic; 2 Department of Animal Science and Food Processing, Faculty of Tropical AgriSciences, Czech University of Life Sciences Prague, Kamýcká 129, 165 21 Prague 6 –Suchdol, Prague, Czech Republic; Università degli Studi di Napoli Federico II, ITALY

## Abstract

Camera trapping with capture-recapture analyses has provided estimates of the abundances of elusive species over the last two decades. Closed capture-recapture models (CR) based on the recognition of individuals and incorporating natural heterogeneity in capture probabilities are considered robust tools; however, closure assumption is often questionable and the use of an M_h_ jackknife estimator may fail in estimations of real abundance when the heterogeneity is high and data is sparse. A novel, spatially explicit capture-recapture (SECR) approach based on the location-specific capture histories of individuals overcomes the limitations of closed models. We applied both methods on a closed population of 16 critically endangered Western Derby elands in the fenced 1,060-ha Fathala reserve, Senegal. We analyzed the data from 30 cameras operating during a 66-day sampling period deployed in two densities in grid and line arrays. We captured and identified all 16 individuals in 962 trap-days. Abundances were estimated in the programs CAPTURE (models M_0_, M_h_ and M_h_ Chao) and R, package secr (basic *Null* and *Finite mixture* models), and compared with the true population size. We specified 66 days as a threshold in which SECR provides an accurate estimate in all trapping designs within the 7-times divergent density from 0.004 to 0.028 camera trap/ha. Both SECR models showed uniform tendency to overestimate abundance when sampling lasted shorter with no major differences between their outputs. Unlike the closed models, SECR performed well in the line patterns, which indicates promising potential for linear sampling of properly defined habitats of non-territorial and identifiable herbivores in dense wooded savanna conditions. The CR models provided reliable estimates in the grid and we confirmed the advantage of M_h_ Chao estimator over M_h_ jackknife when data appeared sparse. We also demonstrated the pooling of trapping occasions with an increase in the capture probabilities, avoiding violation of results.

## Introduction

The size of wild or human-managed populations of animals is a crucial parameter directly involving a wide range of activities, from conservation to commercial strategies.

One popular tool for researchers, mainly in the last two decades, is capture-recapture (CR) analysis of closed animal population parameters, in which data is processed from camera traps. The recognizability of individual animals based on their natural markings is an essential clue for software such as CAPTURE [1] and MARK [2] implementing the Lincoln-Petersen estimator [3] and, more recently, for spatially explicit capture-recapture (SECR) models [4] which overcome the limitations of predecessors. A widely employed non-invasive method which enables estimation of abundances and densities from assumed closed populations was developed for large striped or specked felids, such as tigers [5,6], jaguars [7,8], leopards [9], ocelots [10], pumas [11], cheetahs [12], European lynxes [13], bobcats [14] and margays [15].

Such long-lived, medium- to large-sized mammals are suitable subjects for closed population CR analysis thanks to their potential to meet one of its basic requirements, namely constancy of population size during the study period. Conducting a camera trap study in time periods as short as possible in order to minimize births, deaths and migration should satisfy the closure assumption, in terms of species demography. Regarding geographical closure, especially for felids and other animals with enormous home ranges, attention should be paid to the appropriate spatial design of sampling grids [5,16,17]. Researchers seeking reliable estimates must also take into consideration that the probability of capturing wild-ranging animals may also naturally vary among sampled individuals with regard to their age, social status, sex, fitness, etc. [18], and even among species [19,20]. Heterogeneity will cause underestimation of abundance if a model assuming uniform capture probability is applied [21]. Attempting to cope with defective sources of heterogeneity in capture or detection probabilities, a consensus about the robustness of application of the jackknife estimator, M_h_ [21] predominates in numerous studies [5,8,9,12,22]. However, drawbacks in the accuracy of the estimates, which originate from the small sample size (i.e. few captures and recaptures), were examined [3,16,23,24]. For example, Chao [25] pointed out that the jackknife estimator usually underestimates the population size when data is sparse and proposed modified M_h_ Chao estimator. The results can be biased also because camera traps along the border of deployment could detect animals whose home ranges lie predominantly outside the selected area and which are not representatives of the surveyed population [26]. Additionally, an average capture probability of the sampled animals ($\hat{p}$) lower than 0.1 could severely violate the reliability of the results [16]. As modeled [17], the use of an M_h_ jackknife estimator may result in over- or under-estimations of real abundance when the heterogeneity is high. Several authors [5,10,14] overcame this obstacle and raised the $\hat{p}$ value by pooling capture occasions, which, on the other hand, could theoretically disrupt the assumption of population closure if applied over a long period.

A recent approach which incorporates the location-specific capture histories of marked individuals is the spatially explicit capture-recapture, SECR [4]. The basic assumption is that the source of variability in the detection of individuals is the proximity of a detector to the center of activity. SECR deals with accidental visits along edges of the trapping array, and the estimated density of animals refers to the study area [4]. Likelihood-based SECR modeling allows multiple detections of individuals per trapping occasion, even from polygons or linear transects [27]. Despite widespread use of the method among a scientific audience [28–31], empirical evaluations of its outputs are rare [32–34].

In light of the direct application of abundance estimates in the conservation of wild-ranging animals, we focused on analyzing related sources of bias in a marginalized subject, ungulates. An exemplary species, which manifests white markings that are noticeable, lifelong and unique to each individual, is the Western Derby eland (*Taurotragus d*. *derbianus*), a critically endangered subspecies of one of the world’s largest antelopes (IUCN 2008). In particular, its 10 to 18 vertical stripes, present on each flank in a barcode-like pattern, provide a convenient clue for the identification of individuals from photographs. Images of both flanks are illustrated in taxon’s studbook identification cards [35], which annually list every Derby eland in the semi-captive breeding program in the fenced reserves of Bandia and Fathala in Senegal [36].

Capture-recapture models are surprisingly poorly adopted for herbivores [37]. Instead, ungulates are a common subject of camera-trap surveys which implement relative abundance indices [38–40], which refer to trends and changes in the population rather than to the real size [41]. In this study, we applied for the first time a nonspatial and spatial CR models on a closed population of marked antelope. Our goal was to empirically determine the most appropriate model, which will enable reliable estimates of abundance based on proposed detector array, duration of sampling and density of cameras. We estimated the Derby eland’s abundance using the programs CAPTURE (models M_h_ and M_0_) and R, package secr (basic *Null* and *Finite mixture* models) [42], in two different densities of camera traps in the line and grid placement derived from the x-matrix covering the entire reserve of Fathala. The results, which change with the variable duration of the trapping period, were compared with the known real abundance. We also tested the pooling of trapping occasions and its impact on results and compliance with the closed model assumptions. Our findings will support researchers, conservationists and managers in choosing an appropriate procedure in an effort to estimate the population size of large identifiable ungulates in similar conditions.

## Methods

### Ethics Statement

Our study took place in the private reserve of Fathala with the agreement of the Fathala Tourism Company and the Society for the Protection of the Environment and Fauna in Senegal. We located all cameras strictly on private soil within the fenced area of the reserve. The non-invasive nature of the method neither disturbed the animals nor involved a direct encounter with them. No bait was used.

### Study area and taxon

Our study took place from May to September 2013 in the Fathala reserve, a sanctuary and part of the UNESCO site Delta de Saloum National Park on the western coast of Senegal (GPS coordinates of the main gate are 13°38'27.9"N; 16°25'51.9"W). The vegetation of the sanctuary consisted of Sudano-Guinean savanna with *Andropogon guayanus* and *Pennisetum purpureum* dominating in the undergrowth [43]. The largely flat topography is eroded by one wadi, a seasonal river valley crossing the northern part of the reserve in an east-west direction. The wadi contains running superficial water only at the peak of the rainy season, which lasts from July to September.

The fenced 10.6-km^2^ section of the reserve was occupied, together with the Derby eland, by other species of African ungulates (Table 1).

**Table 1:** List of species of ungulates captured by camera traps in the Fathala reserve.

Species	Common name	Order	Family
*Syncerus caffer*	African buffalo	Cetartiodactyla	Bovidae
*Hippotragus equinus*	roan antelope	Cetartiodactyla	Bovidae
*Kobus ellipsiprymnus*	waterbuck	Cetartiodactyla	Bovidae
*Taurotragus oryx*	common eland	Cetartiodactyla	Bovidae
*Tragelaphus scriptus*	bushbuck	Cetartiodactyla	Bovidae
*Taurotragus derbianus*	Derby eland	Cetartiodactyla	Bovidae
*Giraffa camelopardalis*	giraffe	Cetartiodactyla	Giraffidae
*Ceratotherium simum*	white rhinoceros	Perissodactyla	Rhinocerotidae
*Equus quagga*	plain zebra	Perissodactyla	Equidae

**Fig 1:**
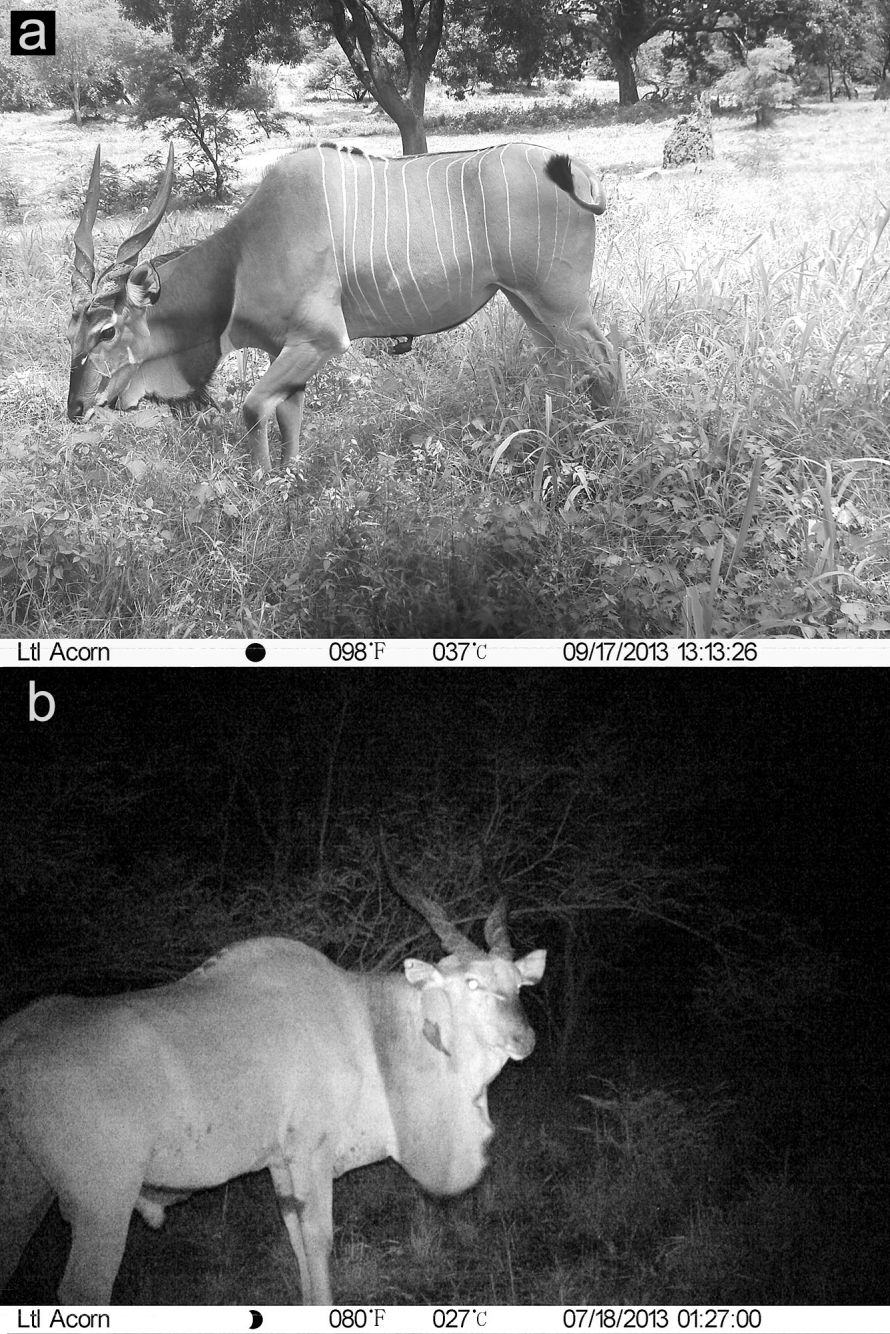
Photographs of a Derby eland female in the daytime (a) and a male at night (b) taken by camera traps in the Fathala reserve, illustrating the poor visibility of markings in the night shot.

Altogether, 16 Derby elands (real density = 1.51 animals per 1 km^2^) inhabited the studied section during the entire trapping period. The population consisted of 13 adult males, two adult females and one juvenile male. Each animal or part thereof photographed by a camera trap was manually compared with the pattern of white-striped flanks depicted in the African studbook [35]. Following Nežerková [44] we used these morphological criteria in the process of identification: number, position and shape of stripes, white markings on head, dimension and shape of horns (Fig 1). Sex was determined based on external genitalia and dimensions of horns. Two observers (T. J. and K. H.) independently analyzed all the images, and only consensual identifications were included in the dataset.

### Camera trapping tools and design

**Fig 2:**
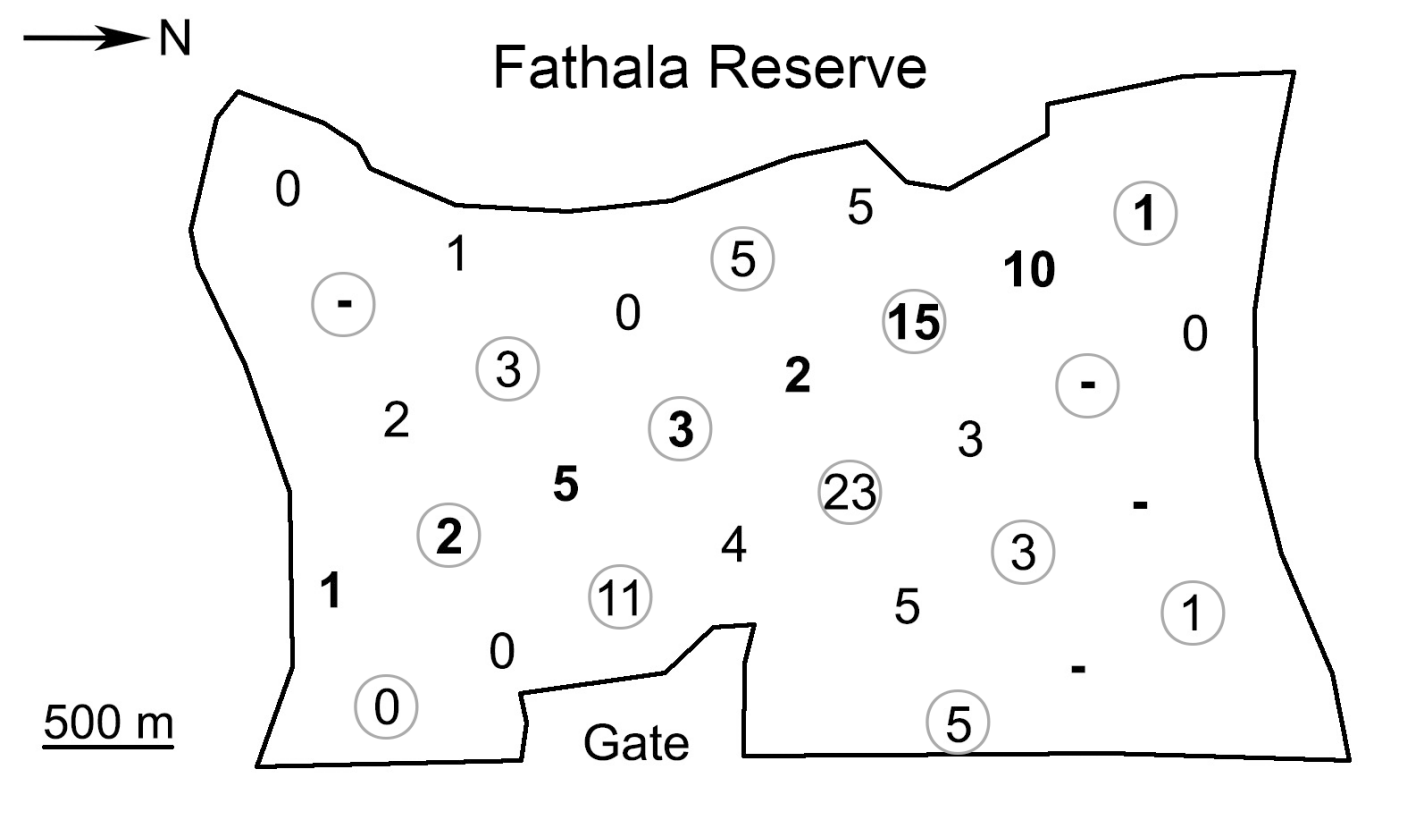
Map of camera trap placement in the Fathala reserve during the sampling period, showing the number of independent captures of an identified Derby eland by a particular camera trap. Bold numbers highlight the analyzed line of camera traps, circles highlight reduced grid and line; dashes denote malfunctioning cameras.

We used 30 Ltl Acorn 5210MC (Shenzhen Ltl Acorn Electronics Co., Ltd., China) weatherproof infrared (IR) digital camera traps operating in photo mode with a resolution of five megapixels. The IR flash was used in attempt to avoid disturbance of animals, although images taken at night are only back and white and not as readable as those from white flash [45]. Units were placed in a grid with a regular span of 500 m throughout the entire reserve, avoiding facilities and fences. The placement pattern was designed to generate data from a) the entire grid, b) reduced grid of 14 cameras, c) a single line of eight cameras and, d) a reduced line of four camera traps crossing every habitat transversely (Fig 2).

Every final location of a camera trap varied within a 5-meter GPS error from the preliminary defined points, which enabled us to set each trap up to 10 meters from the nearest animal trail in use. Pursuant to findings from our pilot testing of camera traps in the reserve during a 2-week period in February 2013, units were tightened to tree trunks or large bushes between 1.0 and 1.5 m in height. A motion PIR sensor and 52-degree-wide camera lens faced as perpendicular as possible to the trail and north- or southwards to minimize activation of the trigger by direct sunshine. Every camera trap was covered by a flat sheet metal roof as a protection against rain and sun.

Camera trap units were programmed to be in single photo mode with a 0-second interval between two consecutive images which, in the case of the selected model of camera trap, meant a real minimal interval of 6 seconds. Units operated 24 hours a day, and a built-in infrared flash enabled black-and-white photographs to be taken without disturbing the animals. The sensitivity of the motion sensor was set at ‘normal’. Units operated without any maintenance, with a power supply of 8 AA alkaline batteries.

The time schedule was set to allow for at least two months of trapping, beginning on May 11^th^, 2013, before the rainy season. Units were collected on September 21^st^, 2013. We experienced a malfunction of four cameras due to battery leakage. Comparing the minimal lengths of the cameras’ operational period, we were finally able to utilize the data from 26 camera traps for computation in CAPTURE. These devices functioned for 66 days; each day was considered a trapping occasion. In secr, we processed the data from all 30 cameras with application of the *usage* function, which treats the varying detector-specific effort.

### Estimates of abundance

We tested closure of the Derby eland population in the reserve by direct observation of all 16 animals before and after the study, with no change detected.

For estimation of abundance via nonspatial CR analyses, we used the time-tested program CAPTURE. For each recognized Derby eland we generated a capture history, which consisted of a row of 66 numbers, marked 1 if the animal was photographed within the occasion, or 0 if it was not. All available models of the software, differing in assumptions of capture probabilities, were used. In every processed test, the models M_h_ (capture probability differs among animals, usually considered realistic), which use the jackknife estimator, as well as M_0_ (assuming constant capture probability) were determined to be appropriate by CAPTURE’s goodness-of-fit test. Our estimated population size ($\hat{N}$) from both models was reported number of captured animals, standard error of estimate (*SE*), capture probability ($\hat{p})$, coefficient of variation of estimates (*CV* = *SE*[$\hat{N}$]/ $\hat{N}$) and lower and upper values of 95% confidence intervals. The closure test was also processed by CAPTURE.

We computed the estimations of Derby eland abundance ($\hat{N}$) using spatially explicit analyses of density estimates in the R language (version 3.1.2, R Development Core Team, 2014) in the package secr (version 2.9.3, [42]). We employed two models—the *Null* model, where detection is affected only by the use of space, and the 2-class *Finite mixture* model (hereinafter *h2*), which allows for the modeling of variation in detection probability among individuals. For each analysis we compared both models with use of the Akaike Information Criterion (AIC) to test which model is preferable. We defined habitat mask, which span within the borders of the reserve and was composed of the number of detectors corresponding with the analyzed density of camera traps (i.e. 30 or 14) with the buffer width of 100 m. For the line arrays, only eight, respectively four cameras were marked as “1” in the secr *usage* argument, remaining 22, reps. 10 had the zero value. The *usage* argument was used also for the determination of every detector’s daily functional state. The detector type for analysis was set as ‘proximity’, which allows multiple detections of individuals on the same occasion. Our sampling area was relatively small and uniform, and we expected the distribution of home range centers to be homogenous. Therefore, distribution was selected as ‘Poisson’. The detection function was equal to half-normal because we assumed that probabilities of capture increase linearly with the proximity of a camera trap to the home range of an individual. We estimated population size ($\hat{N}$) using expected *E (N)* as the volume under a fitted density surface. The value is then equal to the density ($\hat{D}$) multiplied by the area of the region; the standard error is a product of the same equation [42].

## Results

### Identification

**Fig 3:**
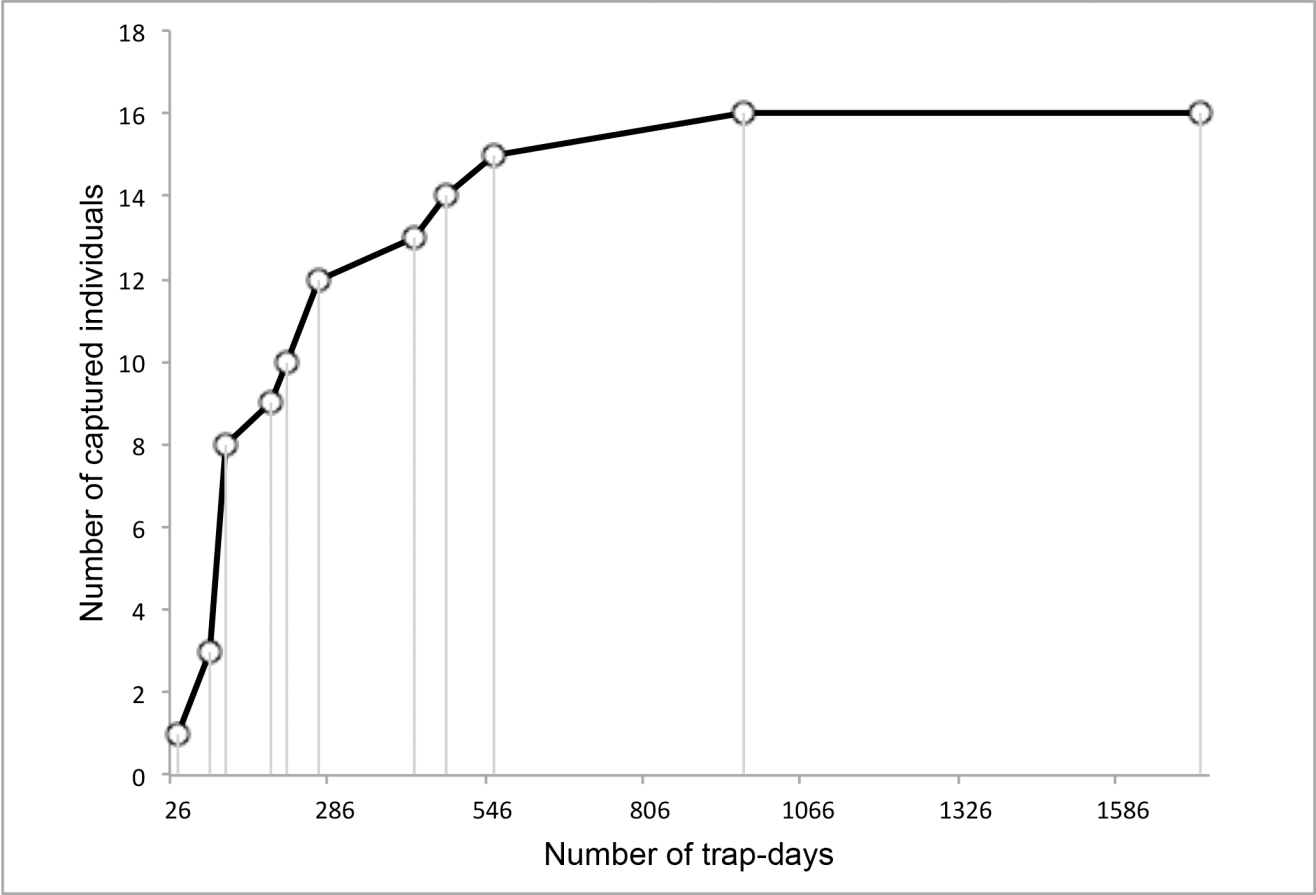
Development of cumulative captures of identified Derby elands in the Fathala reserve.

We accumulated data from a total of 1,716 trap-days from 26 camera traps. During a trapping period of 66 days, our devices took 16,911 photographs, of which 358 (2.1%) were images of Derby elands or parts thereof. We subsequently recognized 192 events of non-identified Derby elands’ encounters with camera trap. Finally, we were able to identify 108 independent captures of 16 Derby eland individuals, scoring a 56.3% success rate in recognition. Hence, the trapping rate was 6.29 (detections of individuals/100 trap-days) and the average trapping effort resulted in 15.9 trap-days per one capture. We needed 962 trap-days to capture and recognize all 16 Derby elands inhabiting the reserve (Fig 3). The first identified animal was photographed in the first day of monitoring, which is equal to 26 trap-days. We successfully distinguished 1 juvenile male, 2 females and 13 adult males.

### Abundance estimates

The assumption of the Derby eland’s population closure during the study period was proven by the goodness-of-fit test in CAPTURE (*z* = -0.382, *P* = 0.351).

**Fig 4:**
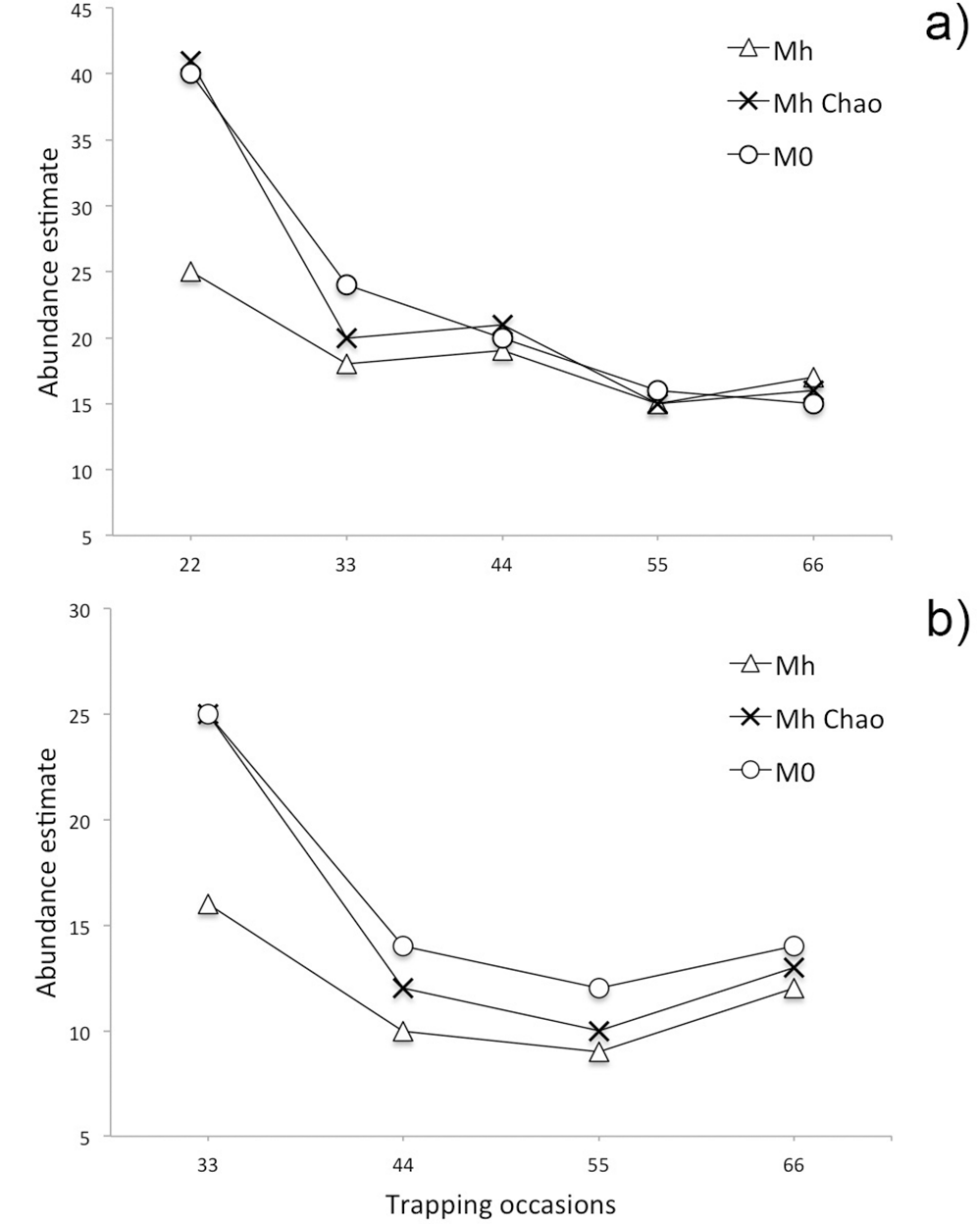
Development of abundance estimates provided by closed (M_0_, M_h_, M_h_ Chao) during 66 trapping occasions in a) the line and b) reduced line camera trap placement.

CAPTURE’s model M_h_ was selected as the most appropriate for every pattern, as it scored 1.00 in the selection criterion, followed by M_0_. The suggested estimator was the jackknife except for the grid and reduced grid at 44 occasions, where M_h_ scored 0.95 and 0.93 after M_0_. The 95% confidence limits of selected models did not include the true value in three cases of M_h_—the grid (44 occasions) and in the reduced grid (55 and 66 occasions) when the lower limit scored identically 17. In the grid, all three models produced the estimated the size of Derby eland population identically 16 animals at 66 occasions with a lowest value of *SE* = 0.15 in M_0_ Chao. In the line pattern for the same trapping period, only M_h_ Chao scored 16 individuals (*SE* = 2.3). As seen in Table 2 and Table 3, the shorter trapping period lasted, the more variable results CAPTURE’s models produced. The sparse data of the shortest periods of both line patterns resulted in higher estimates in M_0_ and noticeably lower in M_h_. Estimator of Chao performed results in between these two models (Fig 4), however, all closed models finally underestimated real size in the reduced line pattern—M_0_ ($\hat{N}$ = 14, *SE* = 2.7), M_h_ ($\hat{N}$ = 12, *SE* = 3.0), M_h_ Chao ($\hat{N}$ = 13, *SE* = 2.2).

**Table 2:** Average capture probabilities ($\hat{p}$) of Derby elands for the full and reduced grid and line placement patterns (CAPTURE, models M_0_, M_h_ and M_h_ Chao).

**Occasions**	**Grid**			**Grid reduced**		
	**M**_**0**_	**M**_**h**_	**M**_**h**_ **Chao**	**M**_**0**_	**M**_**h**_	**M**_**h**_ **Chao**
11	0.113	0.117	0.096	0.113	0.117	0.096
22	0.106	0.105	0.099	0.083	0.078	0.066
33	0.101	0.101	0.101	0.070	0.062	0.070
44	0.097	0.091	0.091	0.076	0.067	0.067
55	0.100	0.100	0.100	0.066	0.062	0.066
66	0.096	0.096	0.096	0.064	0.061	0.064
	**Line**			**Line reduced**		
11	na	na	na	na	na	na
22	0.011	0.018	0.011	na	na	na
33	0.020	0.027	0.024	0.010	0.015	0.010
44	0.023	0.024	0.022	0.017	0.025	0.021
55	0.033	0.035	0.035	0.018	0.024	0.022
66	0.040	0.034	0.036	0.022	0.025	0.023

**Table 3:** Developments of the mean capture probability ($\hat{p}$) of Derby elands depending on the pooling of 66 capture occasions in the line placement patterns (CAPTURE, model M_h,_).

Pooling	Captured	$\hat{N}$	*SE*	$\hat{p}$	95% *CI*
none	14	17	2.6	**0.034**	15–27
2 days	14	17	2.6	**0.066**	15–27
3 days	14	17	2.5	**0.099**	15–26
6 days	14	17	2.4	**0.193**	15–25
11 days	14	17	2.2	**0.304**	15–25

$\hat{N}$ denotes estimated abundance

The M_h_ model’s estimated average probability of capture ($\hat{p}$) reached the verified threshold of reliability 0.1 of the CR estimates only in the grid pattern and at 11 trapping occasions in the reduced grid (Table 2). As mentioned above, low capture probability can be increased by the pooling of capture occasions. Therefore, we undertook a trial computation of the line pattern data pooled out of 2, 3, 6 and 11 days (33, 22, 11 and 6 occasions), resulting in an increase of the parameter $\hat{p}$ from 0.034 to 0.304 (Table 3).

We did not recognize major differences between chosen SECR models outputs. As tested using AIC, the *h2* mixture model was never preferred in each computation. Both models along with rising sampling period consistently decreased their initially overestimated abundances to the nearly real size value. The models in the grid at 66 occasions scored equally $\hat{N}$ = 16.1, *SE* = 4.1, in the reduced line the *h2* model was slightly more precise ($\hat{N}$ = 15.4, *SE* = 5.4) than the *Null* model ($\hat{N}$ = 15. 1, *SE* = 5.3). Generally, the *h2* model performed similarly better when data appeared sparse (Tables 4 and 5).

**Table 4:** Estimations of abundance $\left(\hat{N}\right)$ of Derby elands with parameters within different durations of sampling for the full and reduced grid and line placement patterns using *Null* model in secr and M_0_ in CAPTURE.

Grid		SECR *Null*				CAPTURE M_0_			
**Oc.**	***N***	$\hat{N}$	***SE***	***CV***	***95% CI***	$\hat{N}$	***SE***	***CV***	***95% CI***
11	12	**19.1**	6.9	0.36	10–38	**15**	3.3	0.22	12–27
22	15	**16.6**	4.4	0.27	10–28	**16**	1.4	0.09	16–22
33	15	**15.7**	4.1	0.26	9–26	**15**	0.7	0.05	15–15
44	16	**16.4**	4.2	0.25	10–27	**16**	0.4	0.03	16–16
55	16	**16.2**	4.1	0.25	10–26	**16**	0.2	0.01	16–16
66	16	**16.1**	4.1	0.25	10–26	**16**	0.9	0.06	16–16
**Grid reduced**									
11	12	**19.0**	6.8	0.36	10–38	**15**	3.3	0.22	12–27
22	14	**17.8**	5.2	0.29	10–31	**16**	2.1	0.13	15–24
33	15	**17.5**	4.8	0.27	10–30	**16**	1.5	0.09	16–23
44	15	**15.9**	4.2	0.26	10–27	**15**	0.7	0.05	15–15
55	16	**17.0**	4.3	0.26	10–28	**16**	0.7	0.04	16–16
66	16	**16.7**	4.3	0.25	10–27	**16**	0.5	0.03	16–16
**Line**									
11	3	na	na	na	na	na	na	na	na
22	9	**27.2**	19.9	0.73	8–98	**40**	34.5	0.86	15–189
33	12	**22.9**	9.7	0.42	10–51	**24**	9.2	0.38	16–57
44	13	**20.1**	7.0	0.35	10–39	**20**	5.2	0.26	15–38
55	14	**16.9**	4.8	0.29	10–29	**16**	2.2	0.14	15–25
66	14	**15.3**	4.2	0.28	9–26	**15**	1.2	0.08	15–21
**Line reduced**									
11	3	na	na	na	na	na	na	na	na
22	4	na	na	na	na	na	na	na	na
33	8	na	na	na	na	**25**	20.6	0.82	10–115
44	9	**22.0**	13.2	0.60	7–65	**14**	6.1	0.43	10–38
55	9	**19.1**	10.2	0.54	7–51	**12**	4.2	0.35	9–29
66	11	**15.1**	5.3	0.35	8–30	**14**	2.7	0.19	12–24

Oc. denotes number of trapping occasions, *N* is number of captured individuals

**Table 5:** Estimations of abundance $\left(\hat{N}\right)$ of Derby elands with parameters within different durations of sampling for the full and reduced grid and line placement patterns using models allowing for heterogeneity in capture probabilities (*h2*, *Finite mixture* model in secr, M_h_ and M_h_ Chao in CAPTURE).

Grid		SECR *h2*				CAPTURE M_h_				CAPTURE M_h_ Chao			
**Oc.**	***N***	$\hat{N}$	***SE***	***CV***	**95% *CI***	$\hat{N}$	***SE***	***CV***	**95% *CI***	$\hat{N}$	***SE***	***CV***	**95% *CI***
11	12	**19.1**	6.9	0.36	10–38	**14**	3.6	0.25	12–29	**17**	6.5	0.38	13–44
22	15	**17.0**	4.6	0.27	10–29	**16**	2.9	0.18	16–34	**17**	2.6	0.16	16–29
33	15	**15.7**	4.1	0.26	9–26	**15**	3.8	0.25	15–15	**15**	0.0	0.00	15–15
44	16	**16.4**	4.2	0.25	10–27	**17**	1.5	0.09	17–24	**17**	1.3	0.08	16–24
55	16	**16.2**	4.1	0.25	10–26	**16**	2.1	0.13	16–16	**16**	0.0	0.00	16–16
66	16	**16.1**	4.1	0.25	10–26	**16**	0.9	0.06	16–16	**16**	0.0	0.00	16–16
**Grid reduced **													
11	12	**18.8**	6.8	0.36	10–37	**14**	3.6	0.25	12–29	**17**	6.5	0.38	13–44
22	14	**17.7**	5.1	0.29	10–31	**17**	3.6	0.21	15–32	**20**	6.5	0.32	16–47
33	15	**17.6**	4.8	0.27	10–30	**18**	2.6	0.15	16–28	**16**	1.5	0.09	15–23
44	15	**16.1**	4.3	0.26	10–27	**17**	2.4	0.14	16–27	**17**	3.7	0.22	16–37
55	16	**17.0**	4.3	0.26	10–28	**17**	1.7	0.10	17–25	**16**	0.5	0.03	16–19
66	16	**16.6**	4.2	0.25	10–27	**17**	4.2	0.25	17–44	**16**	0.5	0.03	16–19
**Line**													
11	3	na	na	na	na	na	na	na	na	na	na	na	na
22	9	**27.2**	19.9	0.73	8–98	**25**	8.9	0.36	15–53	**41**	39.6	0.97	14–220
33	12	**23.2**	9.9	0.43	10–52	**18**	4.6	0.25	14–34	**20**	7.5	0.37	14–49
44	13	**20.5**	7.1	0.35	11–40	**19**	4.6	0.24	15–35	**21**	7.5	0.36	15–50
55	14	**17.8**	5.4	0.31	10–32	**15**	7.1	0.48	15–63	**15**	1.5	0.10	15–22
66	14	**15.5**	5.1	0.33	8–29	**17**	2.6	0.15	15–27	**16**	2.3	0.14	15–26
**Line reduced **													
11	3	na	na	na	na	na	na	na	na	na	na	na	na
22	4	na	na	na	na	na	na	na	na	na	na	na	na
33	8	na	na	na	na	**16**	6.3	0.39	10–38	**25**	23.6	0.94	10–134
44	9	**22.1**	12.9	0.58	8–64	**10**	3.4	0.34	9–26	**12**	4.9	0.41	9–33
55	9	**18.7**	9.4	0.50	7–47	**9**	3.0	0.34	9–27	**10**	2.6	0.26	9–22
66	11	**15.4**	5.4	0.35	8–30	**12**	3.0	0.25	12–30	**13**	2.2	0.17	12–22

Oc. denotes number of trapping occasions, *N* is number of captured individuals

**Fig 5:**
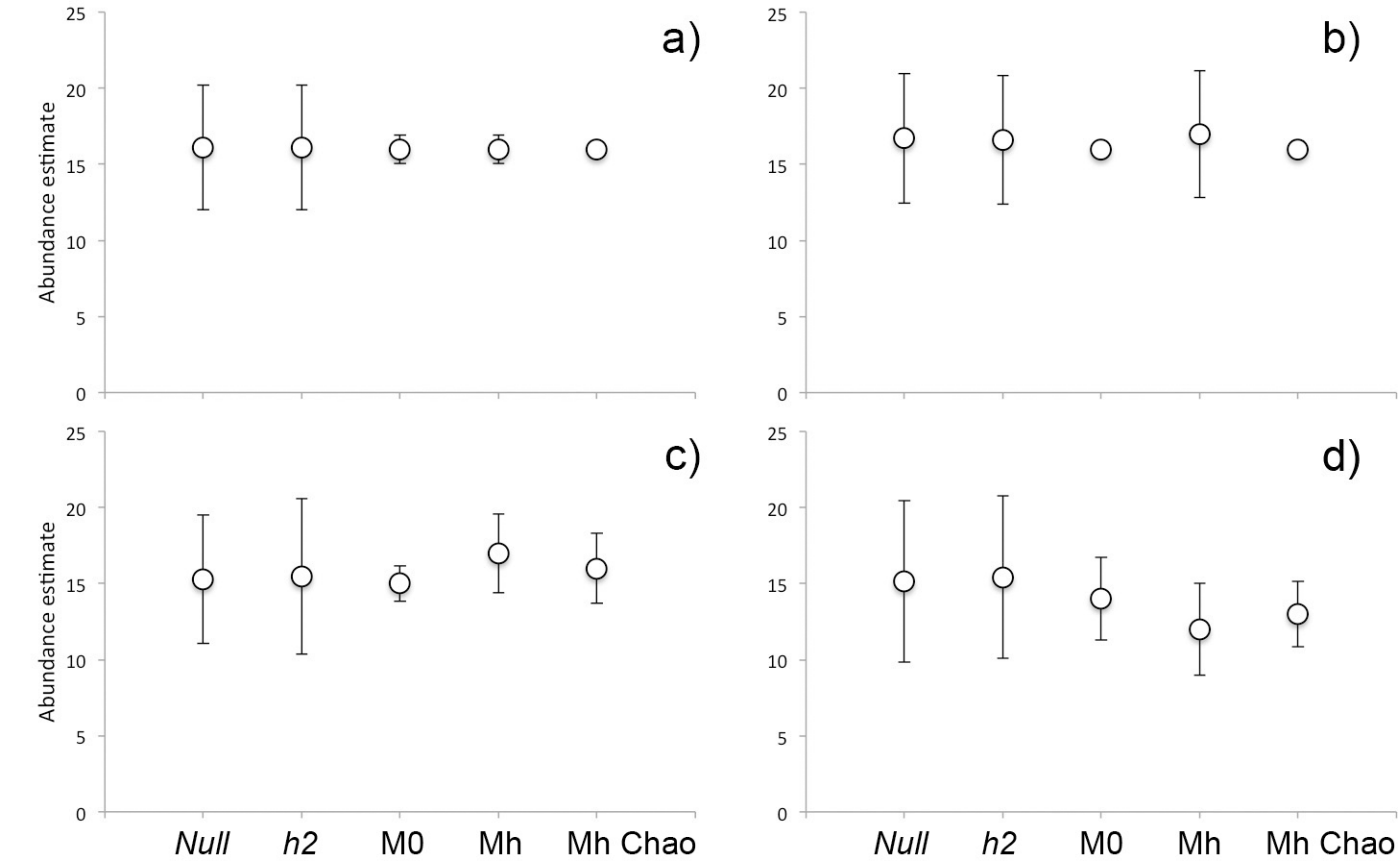
Comparison of abundance estimates $\left(\hat{N}\right)$ of Derby elands in the Fathala reserve gained from spatially explicit capture-recapture (*Null* and *h2* models) and closed CR models (M_0_, M_h_, Mh Chao) at 66 trapping occasions in a) the grid, b) reduced grid, c) line and, d) reduced line camera trap placement. Whiskers denote standard errors.

Detailed parameters of estimates for 11 to 66 trapping occasions in the CR and SECR models are shown in Tables 4 and 5. With the increasing duration of sampling, standard errors decreased with narrowing confidence intervals in all models and arrays (with one exception of M_h_ at 66 occasions in the reduced grid). Our test specified 66 days (1,716 trap-days) as the sufficient period for appropriate abundance estimation in the grid and line pattern regardless of the detector/animal ratio. Mainly SECR, but also CR models reached almost the real size of 16 Derby elands no matter the ratio was 1.88 (grid) or 0.25 (reduced line) (Fig 5).

## Discussion

Our results demonstrated the successful application of camera-trapping for the identification of Western Derby eland individuals. However, we found that the infrared mode for pictures taken at night did not provide clear results. We needed 37 days (962 trap-days) to identify all 16 Derby elands at a density nearly one-hundred times higher than could be expected [46] in the taxon’s last stronghold, Niokolo Koba National Park in Senegal, an endangered UNESCO World Heritage Site. The black-and-white photographs made the distinction of white stripes on the pale fur of the antelope difficult. Hence, only daytime images could be examined. Application of the selected camera traps will constrain the sampling of free-ranging animals which tend to be nocturnal or crepuscular.

The solution would comprise the employment of camera traps equipped with a white flash. Karanth [5], using a white flash in Nagarahole, India, captured 10 tigers in 387 trap-nights with an estimated density of about 0.14 tigers per 1 km^2^. We achieved the same number of Derby elands in 208 trap-days but with a real density of 1.51 individuals per 1 km^2^. Nevertheless, negative behavioral responses, such as the potential avoidance of camera traps [47], should first be tested for a particular taxon and location. The invisibility to humans of the infrared light spectrum protects camera traps from theft, which is not a trivial argument in areas accessible by the public. Researchers must also take into consideration the duration of recharging of the white flashbulb, which constrains the trigger speed of the camera, and discharging of the power supply. This is an important fact, considering that 97.9% of the images taken depicted other species of animals living in the Fathala reserve. Cameras were also triggered by moving vegetation waving in the wind, or by flying insects such as unidentified species of butterflies, moths, termites or flies, and even by spiders hiding in the proximity of the trap’s sensors.

We benefited from the knowledge of the Derby elands flank patterns listed in the identification cards in the African studbook [35]. This economized on material costs because instead of being in pairs, standard in surveys of large cats [5,8,20], cameras could be set in a single placement only and the process of identification was faster.

We successfully tested the accuracy of CR and SECR estimates for the chosen distinctive species of large antelope in conditions of dense wooded savanna in West Africa. Both methods were sensitive to the duration of trapping, hence, the sparsity of capture-recapture data [25]. Unlike the CR models, SECR provided a consistent decline of estimates to the true value. In grid patterns, all models performed well relatively soon, in 22 days, but cameras already caught almost all Derby elands within this period. In the line arrays, where only part of the population was recorded, the poorer data meant for CR model both over- and under-estimation, whereas SECR models showed the same declining pattern with almost no differences between *Null* and *h2* model. The period of 66 days of sampling, which should ensure the closure assumption in the case of large mammals, proved a sufficient time for reliable estimates in all patterns for the SECR. The CAPTURE did not reach the true abundance yet in the reduced line. We can conclude, that spatially explicit models served reliably even within the range of density 0.004 to 0.028 camera trap/ha, or 7.5-times divergent ratio of number of camera traps per one Derby eland. This is a crucial argument because if we are able to properly model the area of habitat of the focal taxon, and we expect its rough density, the calculation of our trapping effort, design and costs is hereby intuitive. Despite being promising, the suggested method requires further examination, mainly in terms of the spatial and temporal distribution of target species and sources.

Use of the jackknife estimator in CR analyses is an intuitive and widely recommended rote in the literature [5,8,9]. We did not confirm the expected underestimation of abundances with the use of model M_0_, with the only exception in the reduced grid. Conversely, estimates were higher for the shortest trapping occasions especially in the line patterns. As suggested [23,25], poorer and sparse capture data affect the jackknife estimator performance and the estimator of Chao brought better results and higher estimates. Anyway, in the scantiest design (reduced line), M_0_ worked the best, which indicates only minor heterogeneity in Derby eland’s capture probabilities.

As we demonstrated, capture probabilities did not vary among models M_0_, M_h_ and M_h_ Chao within each array. This indicates minor differences in spatial use of the studied area by focal animals as well as strict satisfaction of the closed model assumptions. The increasing trend in capture probabilities (Table 2) confirms the conclusion of Tobler [30] that the only way to improve estimates, besides utilizing more detectors, is to extend the survey period. Unlike the latter authors, we showed that the pooling (or collapsing) of trapping occasions could reasonably influence parameters without leaving the results poorer or more biased.

Based on our findings, the results gained from secr demonstrate a negative bias between the accuracy of abundance estimates and the number of trapping occasions. The *Null* and *Finite mixture* models equally overestimated true values when the number of occasions was lower, particularly in the line arrays. Performed SECR computations confirmed outputs of AIC and CR models, which did not support the use of the model incorporating the variation in detection probabilities (*h2*). Due to the use of IR camera traps, we did not expect even any behavioral response to the detectors.

We did not fulfill the scenario of underestimation of density (and abundance) demonstrated by Gerber and Parmenter [33]. The unmodeled variation in SECR tends to produce outputs that are overly precise and biased [48]. A negative bias has been described when the ranging pattern differs between sexes [30], when spatial resource use affects the movement of animals [49], or when home ranges are asymmetric [50]. To date, no study has estimated the home range size of the Derby eland. With the use of available data of its sister species, the Common eland (*Taurotragus oryx*), an adult male could occupy an area of 6 to 71 km^2^ and a female 34 to 360 km^2^ [51]. Our 10.6 km^2^ study site might therefore be relatively disproportionate to 16 Derby elands and their home ranges. However, we fully satisfied the suggestion of Tobler [30] that the camera polygon for a density study should cover no less than the size of one home range.

We confirmed that the x-matrix placement of camera traps covering the entire sampling area produces accurate outcomes in both the spatial and nonspatial capture-recapture models, even in the case of small-sized populations. Especially for fenced game reserves, where migrants do not violate the closure assumption, the CR model remains a reliable and approachable tool for researchers and managers, however old-fashioned it may be. We highlight the potential of the line pattern, the estimates from which closely reached the real population size, along with adequate capture probability when pooling was applied. However, both the poorer data and line distribution of detectors constrained nonspatial models and the advantages of the secr, which defines the habitat mask, became clear. The linear pattern and the secr models may become more topical for the Western Derby eland and other species inhabiting areas, in fact refuges, geographically restricted in human-populated landscapes as found in Africa [52,53]. With reasonable costs, cameras can span parts of a large area, such as the Niokolo Koba National Park (9,130 km^2^), when set on the most frequented trails and crossing a properly modeled and homogenous area of taxon occurrence, where the density is reasonable [54]. The technique can sample ‘oscillating’ herds of herbivores on a low budget relative to conventional but bias-sensitive counting methods such as aerial census [55,56] or distance sampling [57,58]. West and Central African national parks contend with a lack of funding as well as a fundamental knowledge of the real size of animal populations [44,59–63]. The data obtained would provide a valuable foundation for conservation plans and actions to manage the protected areas.

## Supporting Information

S1 Data Fathala Data.zip. Source data for analysis in CAPTURE and SECR models. (ZIP)
