# Supplementary material for: Application of Spatial and Closed Capture-Recapture Models on Known Population of the Western Derby Eland (Taurotragus derbianus derbianus) in Senegal
Source: PLoS One. 2015 Sep 3;10(9):e0136525. doi: 10.1371/journal.pone.0136525 (PMC4559471; doi:10.1371/journal.pone.0136525)
Supplement: S1 Data — (ZIP) [file pone.0136525.s001.zip › Fathala Data/Fathala_CAPTURE/Fathala_CAPTURE_matrices.docx]

**Grid** (66 occasions)

Mike 000000000100000000000000000001000100000000000000010000000100000000

Gala 000100000000100000000011010000000000000000000000000000000000000010

Dawi 001000001010000000001100000010001000001011001010000000000001000000

Tera 000100000000000000000100000010001000001001000001000010000000000000

Nanu 000000000000000100000100000000000000010000000000000100000000000000

Mang 000100000000000000100100000000100000000110000001000000000000000010

Taib 000101000010000010000000000000000000000000000010000010000001000010

Mata 001001000000000010000000000000000000000000000010100000000000000000

Derb 000000000000000001000000000000010000000000000000000010000000000000

Mans 100000000000100001001000000000000000110000000001000000000001000010

Donm 000000100000000000000100000000000000000100000000000000010001000000

Deed 000000010010000000000100000000000000000100001000000000000001000000

Dara 000000000000000000001000001000010000000000011000000000000001000000

Gaaw 000000000100000000000000000001000010000001000010000000110000000000

Tidi 000100000100001000001000000000000100000000000000010000000000000000

Geor 000000000000000000000000000000000000100000000010001000000000000000

**Reduced grid** (66 occasions)

Mike 000000000100000000000000000001000100000000000000010000000100000000

Gala 000100000000000000000010010000000000000000000000000000000000000010

Dawi 001000001010000000000000000010000000001000001000000000000001000000

Tera 000100000000000000000000000000001000001001000000000000000000000000

Nanu 000000000000000100000000000000000000000000000000000000000000000000

Mang 000100000000000000100000000000100000000110000000000000000000000010

Taib 000101000010000010000000000000000000000000000000000000000001000010

Mata 001001000000000010000000000000000000000000000010000000000000000000

Derb 000000000000000001000000000000010000000000000000000000000000000000

Mans 100000000000100001000000000000000000110000000000000000000001000010

Donm 000000100000000000000100000000000000000100000000000000010001000000

Deed 000000010010000000000100000000000000000100000000000000000000000000

Dara 000000000000000000000000000000010000000000001000000000000000000000

Gaaw 000000000100000000000000000000000010000001000000000000100000000000

Tidi 000100000100001000001000000000000100000000000000010000000000000000

Geor 000000000000000000000000000000000000000000000010001000000000000000

**Line** (66 occasions)

Mike 000000000100000000000000000000000100000000000000000000000100000000

Gala 000000000000100000000001000000000000000000000000000000000000000000

Dawi 000000000000000000000100000010000000000000000000000000000001000000

Tera 000000000000000000000100000000001000001001000001000010000000000000

Nanu 000000000000000000000100000000000000000000000000000100000000000000

Mang 000000000000000000000100000000000000000000000001000000000000000010

Taib 000000000000000000000001000000000000000000000000000010000001000010

Mata 000000000000000000000000000000000000000000000000100000000000000000

Derb 000000000000000000000000000000010000000000000000000010000000000000

Mans 100000000000000000001000000000000000000000000000000000000001000010

Donm 000000100000000000000100000000000000000000000000000000000001000000

Deed 000000000000000000000100000000000000000000000000000000000000000000

Dara 000000000000000000000000000000010000000000000000000000000000000000

Gaaw 000000000000000000000000000000000000000001000010000000110000000000

**Reduced line** (66 occasions)

Mike 000000000100000000000000000000000100000000000000000000000100000000

Dawi 000000000000000000000000000010000000000000000000000000000001000000

Tera 000000000000000000000000000000001000000001000000000000000000000000

Mang 000000000000000000000000000000000000000000000000000000000000000010

Taib 000000000000000000000000000000000000000000000000000000000001000010

Derb 000000000000000000000000000000010000000000000000000000000000000000

Mans 100000000000000000000000000000000000000000000000000000000001000010

Donm 000000100000000000000100000000000000000000000000000000000001000000

Deed 000000000000000000000100000000000000000000000000000000000000000000

Dara 000000000000000000000000000000010000000000000000000000000000000000

Gaaw 000000000000000000000000000000000000000001000000000000100000000000
